# Changes in the Plantar Flexion Torque of the Ankle and in the Morphological Characteristics and Mechanical Properties of the Achilles Tendon after 12-Week Gait Retraining

**DOI:** 10.3390/life10090159

**Published:** 2020-08-22

**Authors:** Liqin Deng, Xini Zhang, Songlin Xiao, Yang Yang, Lu Li, Weijie Fu

**Affiliations:** 1 School of Kinesiology, Shanghai University of Sport, Shanghai 200438, China; 18873286059@163.com (L.D.); zhangxini1129@163.com (X.Z.); xiao_songlin@126.com (S.X.); 18049922807@163.com (Y.Y.); 2 Institute of Sport and Sport Science, University of Freiburg, 79117 Freiburg, Germany; lu.li@sport.uni-freiburg.de or

**Keywords:** gait retraining, Achilles tendon, ankle plantar flexion torque, morphology, mechanical properties

## Abstract

Purpose: Although the Achilles tendon (AT) is the largest and strongest tendon, it remains one of the most vulnerable tendons among elite and recreational runners. The present study aims to explore the effects of 12-week gait retraining (GR) on the plantar flexion torque of the ankle and the morphological and mechanical properties of the AT. Methods: Thirty-four healthy male recreational runners (habitual rearfoot strikers) who never tried to run in minimal shoes were recruited, and the intervention was completed (20 in the GR group vs. 14 in the control (CON) group). The participants in the GR group were asked to run in minimal shoes (INOV-8 BARE-XF 210) provided by the investigators with forefoot strike patterns during the progressive 12-week GR. Meanwhile, the participants in the CON group were instructed to run in their own running shoes, which they were familiar with, with original foot strike patterns and intensities. The morphological properties of the AT, namely, length and cross-sectional area (CSA), were obtained by using an ultrasound device. A dynamometer was utilized simultaneously to measure and calculate the plantar flexion torque of the ankle, the rate of torque development, the peak force of the AT, and the stress and strain of the AT. Results: After 12-week GR, the following results were obtained: (1) A significant time effect in the peak ankle plantarflexion torque was observed (*p* = 0.005), showing a 27.5% increase in the GR group; (2) A significant group effect in the CSA was observed (*p* = 0.027), specifically, the increase in CSA was significantly larger in the GR group than the CON group; (3) A significant time effect in the peak AT force was observed (*p* = 0.005), showing a 27.5% increase in the GR group. Conclusion: The effect of 12 weeks of GR is an increase in AT CSA, plantar flexor muscle strength of the ankle, and peak AT force during a maximal voluntary isometric contraction test. These changes in AT morphology and function could be positive for tendon health and could prevent future AT injury.

## 1. Introduction

Given increased attention on public health, daily life demands for exercise, and the health of individuals, it has been reported that there were up to 45 million recreational runners in the United States alone [1]. Furthermore, the population that is participating in running races has exhibited an increase of 57.8% (from 5 million to 7.9 million participants) around the world [2]. Running has many potential benefits, such as reducing the risk of cardiovascular diseases and promoting physical and mental health [3]. However, studies have shown that up to 79% of long-distance runners are injured in a given year [4]; among injuries, Achilles tendinopathy is one of the main running-related musculoskeletal injuries with a high incidence (9.1%–10.9%) [5].

The Achilles tendon (AT) is the largest tendon in the human body [6]. On the one hand, as the largest elastic structure at the foot-shoe interface, the AT can transfer the force generated by the contraction of the triceps surae to the foot; it can also store and release energy during walking and running [7]. On the other hand, repetitive high and nonhomologous loads [8], insufficient recovery time after overloading [9], and poor ankle muscle strength and flexibility have been reported to be the risk factors of AT injuries [9]. The AT is sensitive to the mechanical environment, and either excessive or insufficient loading is detrimental [8], although stimulating the AT in the optimal range or within the “sweet spot” can promote the anabolic metabolism of collagen fibers to avoid the AT degeneration or chronic injury [10]. Therefore, developing a feasible training program may help improve the mechanical properties of the AT by enhancing the load-bearing capacity, and therefore promote running performance and prevent AT injuries.

Foot strike patterns and gait retraining (GR) have attracted biomechanists, sports medicine physicians, and physical therapists around the world. Running with a forefoot strike pattern (FFS) can reduce the impact force and improve running economy [4,11,12,13]. Meanwhile, loading on the AT likely increases because an increased plantar flexor force is required when individuals run with a FFS [14,15]. The increased repetitive loads could be essential for the AT adaptation and homeostasis among runners [3]. Ahn et al. [16] reported that habitual FFS runners activate their plantar flexor muscles 11% earlier and 10% longer than that of habitual rearfoot strike (RFS) runners. Our preliminary study also showed that the peak force of the AT during the stance phase significantly increases after the 12-week GR [9]. Thus, we further speculated that progressive GR could positively affect the mechanical properties of the AT.

Therefore, this study aims to explore the changes in the plantar flexion torque of the ankle, the rate of torque development, and the morphological characteristics and mechanical properties of the AT after 12-week GR. We hypothesized that plantar flexion torque, rate of torque development, cross-sectional area (CSA), and peak force of the AT, stress, and strain would significantly increase in the GR group after a 12-week GR intervention as compared with those in a control (CON) group.

## 2. Methods

### 2.1. Participants

Forty-two participants (age 23.7 ± 2.7 years, height 178.3 ± 2.5 cm, body mass 70.1 ± 4.6 kg) were recruited and randomly divided into GR and CON groups. G*Power (Version 3.1.9.6, Kiel University, Kiel, Germany) was used to calculate the sample size at a power of 0.80, an effect size of 0.25 (medium effect of ANOVA), and a significance level of 0.05 [17]. The required minimal sample size of the prior estimation of two-way repeated ANOVA was 34. The inclusion criteria of the participants were as follows: (1) male recreational runners with habitual RFS who never tried to run in minimalist shoes with FFS, (2) no musculoskeletal injuries of the lower extremity and no neuromuscular diseases in the last 3 months, and (3) a weekly running distance of over 15 km. All the participants provided written informed consent approved by the ethics committee of the University (no. 2017007).

Overall, 34 out of the 42 participants completed the 12-week GR intervention (Table 1). Specifically, one participant who ran with an FFS technique before the training was excluded. During the intervention, one participant quit because they were accidentally injured, i.e., acute ankle sprain. Four runners who were absent from the training for more than one week were also excluded. The reason for their absence was that they were out of contact or had other obligations. Two other participants were excluded because of the mismatch between the training dairy and the cloud data from the app (training duration, frequency, foot strike pattern, etc.) In addition, no participants reported pain in the AT during training.

### 2.2. Instrumentations

**Figure 1:**
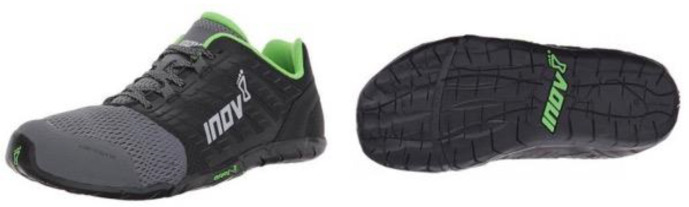
INOV-8 BARE-XF 210 minimalist shoes.

An M7 Super ultrasonography system (Mindray, ShenZhen, China) with a linear array probe was used to measure the CSA, length at rest, and elongation of the AT. A dynamometer (Con-Trex Mj, Schnaittach, Germany) was utilized to determine the continuous plantar flexion torque during maximal voluntary isometric contraction (MVIC). During the training sessions, INOV-8 BARE-XF 210 minimalist shoes (average mass of 227 g, 3 mm total sole thickness, no midsole, and zero heel-to-toe drop, Figure 1) were worn by the participants in the GR group. The inserted Podoon© pressure-sensitive intelligent shoe pads (thickness of 3 mm) that were connected to an app were utilized to monitor the foot strike pattern of the participants. RFS was defined as when the sensor at the heel was triggered two frames early than the sensors at the metatarsophalangeal joint according to the instruction of Podoon© pressure-sensitive intelligent shoe pads. Meanwhile, this shoe pad was also used to replace the original insole of the minimal shoes (same thickness). Furthermore, no participant reported uncomfortable feelings after wearing minimal shoes with changed insoles.

### 2.3. Procedures

**Figure 2:**
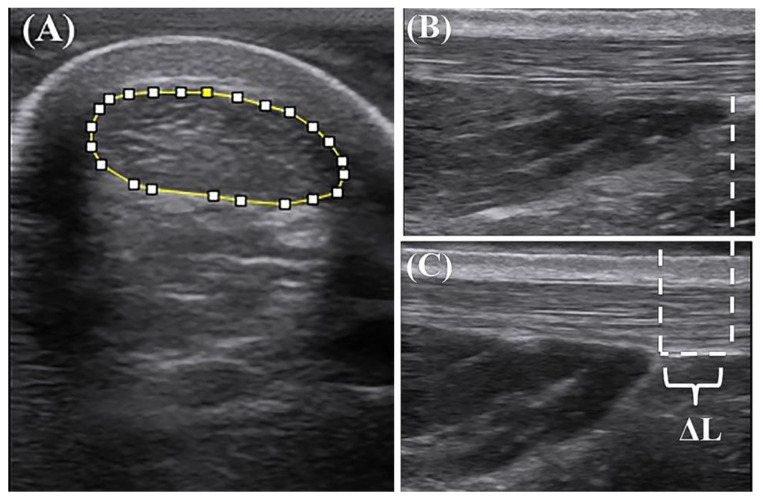
(**A**) Ultrasound image of the cross-sectional area; (**B**) Achilles-soleus myotendinous junction at rest; (**C**) Achilles-soleus myotendinous junction at 100% maximum voluntary isometric contraction. ΔL, the change in the length of the proximal Achilles tendon during the maximum plantar flexion isometric contraction.

During warm-up, the participant wore uniform running shoes (NIKE AIR ZOOM PEGASUS 34) and jogged on the treadmill with a self-selected foot strike pattern at 12 km/h, for 5 min. Meantime, the intelligent shoe pads were used to determine the foot strike pattern. After that, the participants were instructed to prostrate on a treatment table with their ankles in a neutral position (the shank perpendicular to the foot) [9]. The AT length and the CSA at 10 cm above the calcaneus were determined by a single investigator, who was also the same investigator (Figure 2A). Next, the participants were asked to sit on the dynamometer with their ankle in the neutral position, knee fully extended, thigh fixed, and hip against the back of the seat. They were instructed to perform a MVIC. Each test was conducted for 5 s and repeated thrice [9]. During the MVIC, the plantar flexion torque was determined simultaneously with the image of the AT elongation (the distance between the Achilles-soleus myotendinous junction at rest and the same junction at the MVIC, Figure 2B,C). Then, the participants were randomly divided into the GR and CON groups and trained depending on the protocol. After the completion of a 12-week training intervention, the above tests were executed again.

### 2.4. GR Intervention

For the GR group, the participants were asked to wear minimalist shoes and ran outdoor with the FFS at a moderate self-selected speed during training. The FFS referred to the initial metatarsal ball of the forefoot contact followed by the contact of the rest of the foot [15]; in this running style, the feet were placed below the hip during ground contact [18]. The training intervention lasted for 12 weeks, and the training sessions were executed three times per week. The training duration of each training session was increased progressively. Specifically, the training duration of the initial week was 5 min per time. The training duration increased gradually from 5 min per time in 1st week to 48 min per time in the 1st week. Subsequently, it increased by 2 min per time a week from the 8th week to the 12th week, for each training session [19]. The total weekly running distance was consistent with that of the previous training, and the training program only replaced a part of the total amount of running distance. They had to complete the remaining running distance outside of training with their own habitual shoes in old style. During the GR intervention, foot and ankle exercises (i.e., heel raises, short foot exercise, and towel curl exercise) were encouraged to be conducted before each GR session as a warm-up exercise to prevent injuries for participants in the GR group. The detailed training plan and supervision methods can be found in our previous studies [18,19].

For the CON group, the participants were asked to run in their own running shoes with the original foot strike patterns and intensities. To make the running volume comparable with the GR group, the CON group only needed to keep their original running habits (foot strike pattern) and running volume (running intensity and frequency).

### 2.5. Data Analysis

**Figure 3:**
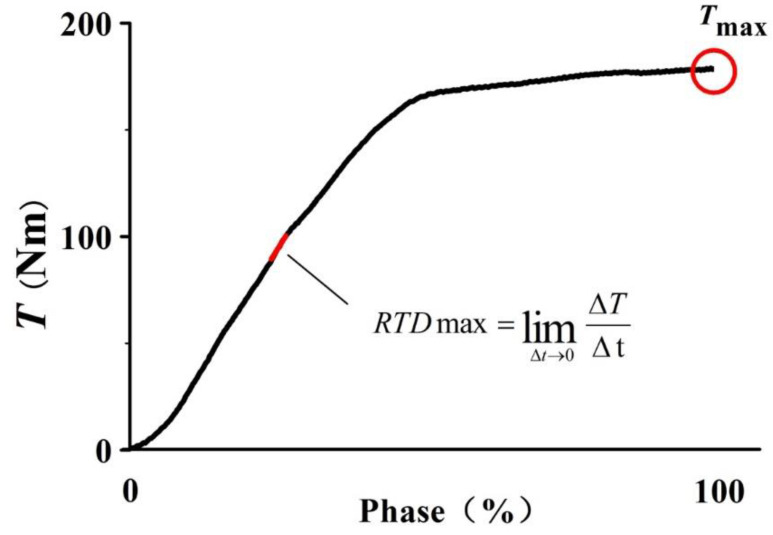
Peak plantar flexion torque-time curve and torque development rate. RTD_max_, the peak rate of toque development; T, the plantar flexion torque; T_max_, the peak plantar flexion torque; ΔT, the change in the peak plantar flexion; and Δt, the time interval.

The plantar flexion torque of the ankle and the peak plantar flexion torque (T_max_) were directly collected from the dynamometer, and then normalized to body weight (dividing by the body weight) [9]. The peak rate of the plantar flexion torque development (RTD_max_, Figure 3) was obtained by calculating the maximal slope of the plantar flexion torque-time curve, namely, the peak first derivative of the plantar flexion torque [20].

The CSA image of the AT was calculated via the Image J software (Version 1.53, NIH, Bethesda, MD, USA) (Figure 2A) [21]. The AT length at rest (L_AT_) was determined in terms of the distance between the AT insertion and the Achilles-soleus myotendinous junction [9]. The elongation of AT (ΔL) was the change in the length of the Achilles-soleus myotendinous junction from at rest to MVIC, which was measured in Image J (Figure 2B,C).

The maximum value was regarded as the peak force of the AT. The peak plantar flexion torque was divided by the AT torque arm to calculate the force of the AT as:

F_AT_ = T_max_/TA_AT_,

where F_AT_ is the force of the AT which was normalized to body weight, and TA_AT_ is the AT torque arm (0.05 m), a default value described by Komi et al. [21,22].

The AT stress was calculated by dividing the peak force of AT by the AT CSA [9] as:

AT stress (σ) = F_AT_/CSA.

The AT strain was calculated by dividing the elongation of AT by the AT length at rest [23] as follows:

AT strain (ε) = ΔL/L_AT_.

### 2.6. Statistics

Shapiro–Wilk tests were used to examine the normality of the variables. If normal distribution was not observed, a Scheirer–Ray–Hare test was performed. Otherwise, a two-way (time × group) repeated measure ANOVA was executed in SPSS (IBM, version 21.0, Armonk, NY, USA) to analyze the effect of 12-week GR on the T_max_, RTD_max_, AT CSA, L_AT_, ΔL, peak force of the AT, stress, and strain. When an interaction between time and group was observed, a simple effect was applied as post hoc analysis. All results were presented as mean ± standard deviation. The significance level was set at 0.05.

## 3. Results

### 3.1. Ankle Torque during MVIC

**Figure 4:**
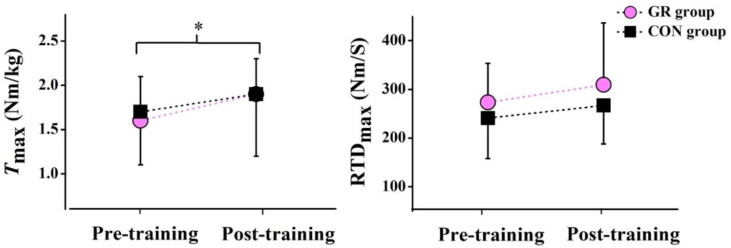
Effects of the 12-week gait retraining on the peak plantar flexion torque (T_max_) and the rate of plantar flexion torque development (RTD_max_). * indicates a significant difference before and after the training (*p* < 0.05). GR, gait retraining and CON, control.

After the 12-week GR, no significant interaction effect between time and group was observed for T_max_ and RTD_max_ during the MVIC. However, there was a significant time effect for T_max_ (*p* = 0.005), showing a 27.5% increase in the GR group and a 10.4% increase in the CON group, after the 12-week intervention (Figure 4). There was no significant main effect of group or time was for RTD_max_ (Table 2).

### 3.2. AT Morphology

**Figure 5:**
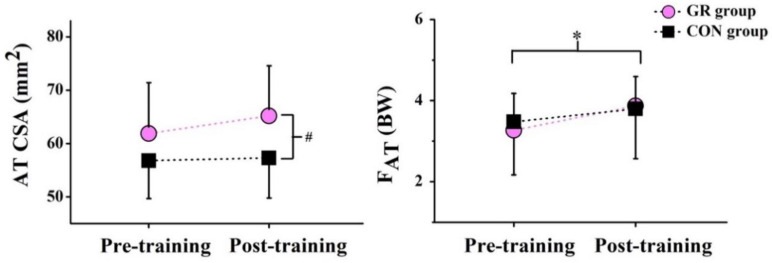
Effects of the 12-week gait retraining on the cross-sectional area of the Achilles tendon (AT CSA) and the peak Achilles tendon force (F_AT_). * indicates a significant difference before and after the training (*p* < 0.05) and # indicates a significant difference between the two groups (*p* < 0.05). GR, gait retraining; CON, control; and BW, body weight.

After the 12-week GR, no significant interaction effect between time and group was observed for all parameters, including CSA, L_AT_, and ΔL. However, a significant group effect in the CSA was observed (*p* = 0.027). Specifically, the CSA was significantly larger in the GR group than in the CON group (Figure 5), showing a 6.3% increase after the GR. There was no significant main effect of group or time detected in L_AT_ and ΔL (Table 2).

### 3.3. AT Mechanical Properties

**Figure 6:**
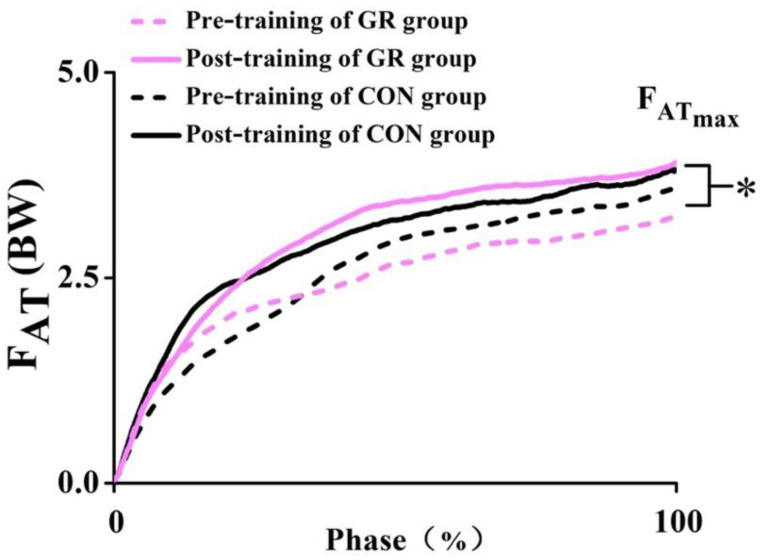
Force-time curve of the Achilles tendon during maximal voluntary isometric contraction before and after the training. * indicates a significant difference before and after the training (*p* < 0.05). GR, gait retraining; CON, control; F_ATmax_, the peak Achilles tendon force; F_AT_, Achilles tendon force; and BW, body weight.

There were no significant interaction effects observed between time and group in force of the AT, stress, and strain after the 12-week GR. However, a significant time effect in the peak force of the AT was observed (*p* = 0.005) (Figure 6), showing a 27.5% increase in the GR group and a 10.4% increase in the CON group (Figure 5). There were no significant main effects for group or time observed in AT stress and strain (Table 2).

## 4. Discussion

The present study aims to explore the effects of 12-week GR on the plantar flexion torque of the ankle and the morphological and mechanical properties of the AT. After 12-week GR, the force of the AT and plantar flexion torque during an MVIC test increased significantly. In addition, the increase in AT CSA in the GR group was larger than that in the CON. This study adds evidence to the idea that changing gait from an RFS to an FFS can strengthen the AT. The implications for injury prevention must be studied.

After the 12-week GR, the plantar flexion torque of the ankle and the peak force of the AT significantly increased for the MVIC test. Specifically, a 27.5% increase was observed in the GR group, whereas a 10.4% increase was found in the CON group. These results were similar to previous findings. For example, Lieb et al. [24,25] reported that the plantar flexion torque and force of the AT during MVICs in habitual FFS runners were significantly higher than those in habitual RFS runners. Meanwhile, Perl et al. [26] found that the plantar flexor force significantly increased during running after a 6-month GR. Similarly, Joseph et al. [14] observed a significant increase in AT force during the MVIC test and CSA after their participants ran in minimalist shoes with an FFS pattern and an increased stride frequency. These findings can be explained by the fact that a greater plantar flexion torque is required by the triceps surae muscles to prevent the collapse of the lower extremities during running with FFS [4]. Although F_AT_ was calculated from T_max_ via a constant factor of TA_AT_ for all participants, the meaning of the T_max_ and F_AT_ was different. T_max_ reflects the strength of the plantar flexor, while F_AT_ represent the magnitude of the mechanical stimulus loaded on the AT. It has been reported that within a reasonable range, a high load can positively affect the mechanical properties of the AT for adaptation [27]. Consequently, the adaptation was accelerated. Furthermore, weak triceps surae muscle strength is one of the risk factors of AT injuries [28,29,30]. The increased plantar flexion torque found in this study indicates increased plantar flexor strength and could decrease the risk of future AT injury.

As one of the indicators for evaluating explosiveness, the rate of torque development is highly and positively related to the performance of running and jumping [31]. Our finding showed that the rate of torque development increased after the training (*p* = 0.074). The triceps surae muscles experienced a high amount of force while FFS was being used [4], potentially increasing the rate of torque development. For long-distance runners, the increased rate of torque development is conducive to the explosiveness of the plantar flexor in the toe-off phase while running. Meanwhile, the AT is the key structure that transmits the strength of the triceps surae. It has been suggested that the AT is able to more efficiently deliver the greater force supplied from stronger triceps surae [32]. Therefore, with the increased plantar flexion torque and rate of torque development, we conjecture the AT could more efficiently transmit muscle strength to push off after the 12-week GR.

The AT CSA of runners is larger that of nonrunners [33]. This may be attributed to adaptive hypertrophy, caused by increased collagen turnover induced by running [34]. In our study, the increase in AT CSA in the GR group was larger than that in the CON group. It must be noted that the AT CSA was already larger in the GR group before the 12-week training program started. This finding was similar to the results of Histen et al. [21], who reported that the AT CSA was significantly greater in runners who previously wore minimalist shoes (self-reported either FFS or a midfoot strike pattern) than runners who previously wore traditional running shoes (self-reported RFS). These findings could indicate that such repetitive increased loads on runners with FFS cause adaptive hypertrophy of the AT [35]. In the present study, there was an increased trend in CSA, although it was not mainly affected by time (*p* = 0.081), that is, a 6.3% increase in the CSA was found in the GR group, and only a 1% increase was detected in the CON group. This result indicated that the CSA increased, but this increase was not significant after the 12-week GR. The nonsignificant change could be attributed to training intensities. In other words, with longer time and greater training intensities greater changes in CSA can be achieved [9]. This inference was confirmed by a series of studies conducted by Arampatzis et al. [27,34] who focused on the effects of isometric contraction training on the AT.

As an indicator of forces exerted on a unit area, AT stress did not change significantly after the 12-week GR; this finding was consistent with previous results [14]. The changes were not significant mainly because the peak force of the AT increased significantly, whereas the CSA increased but insignificantly. On the one hand, this finding indicated that a significant force on the AT could be loaded with an increased CSA after the 12-week GR and under a given stress condition, that is, the load-bearing capacity of the AT was enhanced. Similarly, no significant change was observed in the AT strain. Since the AT stores or releases energy by lengthening or shortening to transmit the force [36], the significant increase in the force of the AT and the nonsignificant changes in the strain indicates that the AT could store more force without increasing the strain. On the other hand, previous studies have demonstrated that excessive stress and strain are important risk factors of AT injury [25], which could cause the degradation of the AT and the microrupture of the AT collagen fibers [37]. In the present study, no significant changes were observed in AT stress and strain. This result suggests that the 12-week GR that we used did not lead to excessive stress and strain, as no AT injuries were encountered.

This study had several limitations. First, the GR group and the CON group were not the same at the start of the study for the outcome CSA measurement. In future studies, this must be controlled. Second, in this study, all participants had the same GR program. In future studies, which training program promotes AT CSA and the mechanical properties of the AT most should be determined, as well as which training program is too strenuous and causes injuries through the use of various training interventions. Third, the participants in the present study were all males; as such, whether females would show the same effects after a 12-week GR remains unclear. Lastly, the long-term retention effects caused by retraining changes were not evaluated in this study.

## 5. Conclusions

In this study, a 12-week GR program led to a 27.5% increase in plantar flexion torque of the ankle and the force of the AT during an MVIC test. The control group showed a 10.4% increase. In addition, the increase in CSA of the AT was larger in the intervention group than in the control group. These results indicate that GR influences the force of the AT and CSA in a positive manner. The implications for tendon health and AT injury prevention must now be studied.

## Figures and Tables

**Table 1:** Basic information of the participants.

Group	Sample Size (*n*)	Age (years)	Height (cm)	Weight (kg)
GR	20	31.3 ± 3.5	175.7 ± 2.6	70.9 ± 3.8
CON	14	26.6 ± 5.8	175.6± 2.3	72.7 ± 4.3

Note: GR, gait retraining and CON, control.

**Table 2:** Effects of 12-week gait retraining (GR) on the plantar flexion torque, Achilles tendon (AT) morphology, and AT mechanical properties.

Parameters	GR Group		Percent Change	CON Group		Percent Change	*p* Value		
	Pre-Training	Post-Training		Pre-Training	Post-Training		Main Effect for Time	Main Effect for Group	Interaction Effect
*T*_max_ (Nm/kg)	1.6 ± 0.5	1.9 ± 0.7 *	27.5%	1.7 ± 0.4	1.9 ± 0.4 *	10.4%	0.005	0.843	0.356
RTD_max_ (Nm/s)	273.5 ± 79.7	309.4 ± 127.0	17.3%	241.0 ± 82.8	267.0 ± 79.1	17.5%	0.074	0.212	0.792
CSA (mm^2^)	61.9 ± 9.5 ^#^	65.2 ± 9.4 ^#^	6.3%	56.8 ± 7.1	57.3 ± 7.5	1.0%	0.081	0.027	0.216
L_AT_ (mm)	77.4 ± 18.1	76.8 ± 18.3	−0.6%	76.9 ± 12.5	77.8 ± 12.2	0.9%	0.776	0.963	0.240
ΔL (mm)	15.9 ± 6.5	18.9 ± 5.2	36.9%	16.4 ± 4.9	16.5 ± 4.7	8.1%	0.166	0.529	0.199
Peak F_AT_ (BW)	3.3 ± 1.1	3.9 ± 1.3 *	27.5%	3.5 ± 0.8	3.8 ± 0.8 ^*^	10.4%	0.005	0.843	0.356
AT stress (MPa)	38.6 ± 13.8	43.0 ± 13.5	20.4%	42.2 ± 11.2	43.9 ± 11.5	5.5%	0.069	0.493	0.191
AT strain (%)	21.9 ± 11.6	25.7 ± 8.7	37.5%	21.9 ± 7.7	21.8 ± 7.0	7.1%	0.234	0.493	0.191

Note: * indicates a significant difference between before and after the training (*p* < 0.05) and ^#^ indicates a significant difference between the two groups (*p* < 0.05). T_max_ is the peak plantar flexion torque, RTD_max_ is the peak rate of plantar flexion torque development, CSA is the cross-sectional area of AT, L_AT_ is the length of AT at rest, ΔL is the elongation of AT, and F_AT_ is the force of AT.
